# Efficacy of Coping with Negative Affect via Alcohol Use Pre- and Post Acute Stress

**DOI:** 10.3390/bs15121614

**Published:** 2025-11-24

**Authors:** Mairéad A. Willis, Tongyao Ran, Sean P. Lane

**Affiliations:** Department of Psychological Sciences, University of Missouri, Columbia, MO 65211, USA; trdvv@missouri.edu

**Keywords:** alcohol use, coping, negative affect, stress

## Abstract

Alcohol use is common for emotional coping due to stress. However, it may not effectively mitigate negative emotions or the stressors. The current study sought to test if use predicts negative affect (NA) relief on days prior to acute stress as predicted by theories such as the stress-response-dampening hypothesis, particularly among those who use to cope. Undergraduates (*N* = 226) preparing for a premedical examination participated in a 14-day diary study (*N_Days_* = 2920). Reports included morning and evening NA, evening alcohol consumption, and a baseline measure of substance-use-directed coping. Multilevel mediation models indicated that only substance-use-directed coping was positively associated with alcohol use on days prior to the exam. In contrast, post-exam, coping was positively associated but morning NA and its interaction with coping were negatively associated with drinking quantity. While drinking was associated with lower prospective evening NA overall, only post-exam indirect effects were observed. NA may not potentiate alcohol use under acute stress, even though use does alleviate NA. Post-stress, NA and substance use coping unexpectedly reduced drinking, perhaps as a protective effort. However, this led to indirect effects whereby NA escalated. Among low/moderate users, the negative reinforcement process is likely not established, though at such levels of use it may be effective.

## 1. Introduction

Although current applications of the evidence-based research domain criteria for alcohol use suggest that negative affect (NA) should both motivate and reinforce use (Al-Khalil et al., 2021), efforts to test this theory have been met with mixed results. Prior literature has investigated (1) the effect of NA on alcohol use (e.g., Hussong et al., 2018; Dora et al., 2023), (2) the effect of stress on alcohol use (e.g., Sher & Levenson, 1982; Croissant & Olbrich, 2004), and (3) the effect of coping motives on alcohol use (e.g., Cooper et al., 2016). 

Coping has sometimes been examined in concert with stress and NA broadly (Bolger & Zuckerman, 1995; Arpin et al., 2025), though less often in scenarios characterized by an acute stressor (Juth et al., 2015; Bolger, 1990; Iida et al., 2017). This is despite foundational literature indicating that differing stress typologies are likely to result in differing impacts (Sinha, 2024; Monroe & Simons, 1991), with a critical distinction between chronic, pervasive stress (e.g., Ansell et al., 2012; Felton & Revenson, 1984) and acute, episodic stress (O’Connor et al., 2021; Bolger & Schilling, 1991; Katz et al., 1981; McGonagle & Kessler, 1990; Rohleder, 2012, 2019).

For alcohol use to mediate the relationship between prior and later NA levels, a significant relationship must exist between NA and alcohol use. Whether this relationship exists and under what circumstances has been debated, but the preponderance of theory and evidence indicates that it does exist on at least some time scale. Studies using retrospective reports have tended to find a relationship between NA and substance use (e.g., depression; Hussong et al., 2018), while a recent large-scale meta-analysis of intensive longitudinal data did not find a consistent relationship between wholesale NA intensity and alcohol use within-days (Dora et al., 2023). Widely accepted theories of substance use behavior, including the empirically-supported addiction cycle, suggest that engaging in substance use should be negatively reinforcing because it relieves withdrawal symptoms, including temporarily reducing NA (Koob & Volkow, 2010).

The affective model of drug motivation (Baker et al., 2004) also suggests that negative reinforcement learning associated with withdrawal may be generalized to other sources of NA, leading individuals to consume alcohol even when alcohol withdrawal is not the cause of NA. To that end, individuals who engage in heavy alcohol use exhibit changes in their stress response that are associated with higher craving (Sinha, 2024), but use may not be as effective at reducing stress as other behaviors that target, and match (e.g., applying the Optimal Matching model outside the social support context; Moon et al., 2019; Cutrona, 1990), the source of distress as opposed to the immediate (or at least relatively local) emotional response (Shu et al., 2021; Iwasaki, 2001).

Many explanations are possible for the apparent lack of relationship between NA and alcohol use at an episodic level, including characteristics of models used to investigate this relationship (Curran & Bauer, 2011), and alternative models of the relationship between affect and alcohol consumption (Baker et al., 2004). Individual differences may account for some of the relationship between stress and alcohol use. The stress-response-dampening hypothesis and extensions of the stress–negative affect model suggest that stress relief following alcohol use may be higher for some individuals, including people at risk for alcohol use disorder (AUD; Croissant & Olbrich, 2004; Hussong & Chassin, 1994). Because negative reinforcement is only theorized to occur once substance dependence is advanced enough to produce withdrawal symptoms (Koob & Volkow, 2010), greater alcohol use in response to NA among people with AUD than among others is a reasonable expectation. However, prior research suggests that individuals at the prodromal stage of AUD risk also experience more physiological stress relief in response to alcohol administration (Sher & Levenson, 1982). Corroborating research indicates that craving relief in response to drinking in youth and young adults ages 15–24 increases with AUD symptomatology (Treloar & Miranda, 2017).

Based on the recent meta-analysis that serves as a counterpoint to the current investigation (Dora et al., 2023), the within-person effect of daily NA on daily drinking when between-person effects are modeled is unclear. This may alter the results if indeed some individuals drink more in response to NA within days than others. Individual differences in motivation to drink and the purpose that drinking serves may partially account for why NA contextually facilitates alcohol consumption, and subsequently reduces NA, compared to when alcohol use is utilized but is ineffective (Walsh et al., 2023).

In addition to consumption driven by NA, the addiction cycle posits two additional stages characterized by different motivators of consumption: anticipation and intoxication (Koob & Volkow, 2010). In the anticipation stage, individuals who have been sensitized to alcohol have increased attentiveness to alcohol-related cues and greater craving (Cofresí et al., 2019). The construct of situation-specific anticipation, or craving, is separable from the construct of enjoyment (Cofresí et al., 2019), which in turn is distinguishable from reduction in NA (Watson et al., 1988). It is possible that this incentive salience sensitization process, or other components of evidence-based models, may be critical antecedents that behaviorally motivate alcohol use as well as precipitating negative affectivity.

Stress is an important contextual factor in explaining the relationship between NA and alcohol use. Life stressors increase risk for AUD (Keyes et al., 2012). Retrospective reports have found substantial support for an effect of life stressors (e.g., Cooper et al., 1992) and even recent stressors (e.g., Stoltzfus & Farkas, 2012; Wills, 1986) on alcohol use. Studies have suggested that among some individuals, alcohol use has a greater stress dampening effect, which may increase the incentive for those individuals to consume alcohol while undergoing stress (Croissant & Olbrich, 2004), though a recent meta-analysis was only able to test one of these moderating variables (family history) and did not find a significant effect (Bresin, 2019).

Even in a sample not targeted for AUD, social drinking has been found to reduce the relationship between stressors and mood and to directly predict (i.e., mitigate) NA (Armeli et al., 2003). NA may also be related to aspects of alcohol use other than quantity. For example, in one ecological momentary assessment (EMA) study, rate of drinking, but not amount of drinking, was found to be associated with reduction in NA (Carpenter & Merrill, 2021).

Motives are an additional factor that may impact the relationship between NA and drinking, given previous findings and their theoretical relationship to alcohol use. That motives would influence the relationship between NA and alcohol use is both theoretically grounded and empirically supported (Cooper et al., 2016; Cox & Klinger, 1988). Predominant theories of addiction, including the motivational model of alcohol use, indicate that individuals will use substances only when they believe that the consequences of use are superior to the consequences of non-use (Cooper et al., 2016; Cox & Klinger, 1988).

Endorsement of coping motives, for example, indicates an optimistic set of beliefs regarding the effects of the substance on affect (Cooper et al., 2016; Cox & Klinger, 1988). In a classic study, Cooper et al. (1992) found that life stressors were associated with alcohol consumption and alcohol-related problems, specifically among men who reported positive alcohol expectancies and men who reported avoidant coping. Since then, the ways that researchers study coping motives has diversified; however, they are anchored by core theories and approaches (Carver & Connor-Smith, 2010; Bolger, 1990; Bolger & Zuckerman, 1995).

In his classic work (Wills, 1986), Thomas Wills found that distraction coping and aggressive coping were positively associated with substance use in a sample of adolescents, with aggressive coping notably predicting substance use more strongly as stress increased. Armeli et al. (2000) did not find an effect of avoidant coping on alcohol use in a study that recorded daily acute stressful events and their subjective severity, but they did find that participant beliefs affected alcohol use. Specifically, men with positive alcohol expectancies drank more on days with higher stress, while men with negative alcohol expectancies drank less (Armeli et al., 2000). Notably, stress did not predict alcohol use among women in this study (Armeli et al., 2000). Likewise, a small (*N* = 32) daily diary study examining effects within weeks found that stress did not predict increased alcohol use among women, but drinking was higher among women with lower problem-focused coping than among women with higher problem-focused coping in times of lower stress (Breslin et al., 1995). This is in contrast to a recent large-scale laboratory study, which found that exposure to an acute stressor predicted alcohol use among women (Patock-Peckham et al., 2022).

Literature on coping and substance use continues to grow. For example, a recent latent class analysis found support for relationships between reactive coping and substance use as well as substance use coping and actual use (Elam et al., 2023). In addition, the same study found further support for a link between aggressive coping and adolescent substance use (Elam et al., 2023). Researchers have also found support for a moderating effect of coping on the relationship between discrimination and substance use (Gerrard et al., 2012). At least one daily diary study found that coping motives mediated a within-person relationship between NA and alcohol-related consequences (Biehler et al., 2024), and another recent study found a relationship between traumatic stress history, coping motives, and alcohol use (Bitsoih et al., 2023).

An important and understudied question in the areas of NA, stress, and alcohol use is whether alcohol use in response to NA, under conditions of acute stress, is an effective means of emotion regulation. In contrast to prediction of alcohol use by NA, prediction of changes in NA by alcohol use has rarely been examined. It is, however, a crucial and testable connection of the hypothesis that alcohol use is negatively reinforcing, as for it to be negatively reinforcing under the terms of most working theories, it must reduce NA in at least some circumstances.

Wycoff et al. (2021), using an ecological momentary assessment design, found that while alcohol use did not predict reduction in reported NA, it did predict subjective current relief. Whether reported NA is reduced after drinking under conditions of acute stress remains unclear. We anticipate that acute stress (pre-exam in the current investigation) will not necessarily translate into escalated alcohol use coping being effective, given the well-established stress and coping matching literature from the field of social support (e.g., Merluzzi et al., 2016; Wang, 2019; Thoits, 1995; Moon et al., 2019; Cutrona, 1990). When instrumental solutions to the source of stress are unavailable, however, using alcohol to cope may be evaluated more positively. For example, in a study of servicemembers, more pessimistic self-appraisal of one’s ability to cope with problems was linked to risky alcohol use (Arpin et al., 2025). For the same reason, alcohol use after the fact, because it myopically and cognitively alleviates individuals’ concerns about uncertain outcomes and may parallel generalized emotional coping concerns under uncertainty, might be emotionally palliative under uncertain coping circumstances (Steele & Josephs, 1990; Cooper et al., 1988, 1992, 1995; Bradford et al., 2013).

### Current Study

The current study seeks to address the following questions: (1) Does higher negative affectivity predict increased alcohol use? (2) Is alcohol use associated with decreased negative affectivity? (3) Does the degree to which individuals endorse alcohol-specific coping to accommodate their distress contribute to lower negative affectivity? Data were collected from undergraduate students who were preparing for a stressful examination across two large metropolitan universities. Across both study locations, we recruited individuals using standardized interview protocols prior to initial inclusion and remunerations as specified in the university Institutional Review Board (IRB) approvals. The study was originally designed to examine the methodological phenomenon known as the initial elevation bias (also known as attenuation effect), in which initial longitudinal assessments appear to be elevated for no reason other than that they are the first administration of a scale (Shrout et al., 2018). The analyses presented herein address independent hypotheses concerning the impact of alcohol use on NA during a period of acute stress, and the effect of coping in motivating alcohol use under such circumstances. First, we predicted that alcohol use would mediate the positive relationship between morning and evening NA, such that morning NA would positively predict alcohol use, which would negatively predict evening NA. Second, we proposed two competing predictions regarding the impact of coping motives on the relationship between alcohol use and negative affect in the context of an acute stressor. On the one hand, research on stress and alcohol expectancies and research on substance use coping support the hypothesis that endorsing substance use coping would predict higher alcohol use in the context of an acute stressor (pre-exam). Alternatively, however, motivation to engage in alcohol use coping might conflict with other goals (e.g., exam preparation) in the presence of a concrete task but might resume once the stressor had passed (post-exam), and students were primarily coping with the uncertainty of awaiting their exam results. We predicted that either of these competing hypotheses could be supported.

## 2. Methods

### 2.1. Participants

Participants were pre-med undergraduate students recruited from New York University (NYU) and Columbia University (CU), with 70% from NYU and 30% from CU. All participants provided informed consent (parent project approval HS#7062 [NYU]), and all data collection procedures were approved by the Institutional Review Boards of the two universities. A total of 246 individuals were initially recruited, of whom 232 passed inclusion criteria, which included being enrolled at NYU or CU and in a pre-medical required course with an upcoming midterm exam. Participants were not required to intend to pursue medical school. Parental consent was required for participants under 18 years of age. Participants completed baseline measures and were randomly assigned to one of seven staggered daily diary conditions (see Shrout et al., 2018). Six additional individuals were excluded because they did not complete at least one full day (morning and evening) of diaries.

In the included current sample of 226 individuals, 71% of the participants identified as women (29% men, 0% another gender identity). Median participant age was 20 years (*M* = 20.04, *SD* = 2.07, *Range* = 18–29) with most in their freshman (32%) or sophomore (34%) year in college. Demographically, 44% of the participants identified as Asian, 43% as White, 7% as Black, 13% as Hispanic (Latine), 28% as international, and 12% as another identity. The pre-med midterm exams participants were preparing for were chemistry (55%), biology (33%), and physics (12%; Table 1).

### 2.2. Study Design

Participants were randomly assigned to one of seven groups and started the diary survey between 8 days and 2 days before their selected exam. They completed a morning and an evening diary for at least 14 consecutive days regardless of the start day. Group 1 was designed to be the comparison group for the other six conditions in the original study (Shrout et al., 2018). It consisted of 31% of participants, who completed 15 twice-daily diary entries to optimize power for contrasts with each of the other 14 twice-daily diary experimental conditions. Most exams were on Fridays (69%), followed by Thursdays (20%), Mondays (5%), Wednesdays (5%), and Tuesdays (1%). At the morning waking time report, participants completed morning diary surveys online through an emailed web link using a personal device (i.e., computer, tablet, or smartphone); evening diaries were completed via the same method before the participant went to bed. For more study design information, refer to Shrout et al. (2018).

### 2.3. Measures

#### 2.3.1. Substance Use Coping

Substance use coping was measured at baseline using an item adapted from the Alcohol-drug disengagement subscale derived from the revised COPE (Zuckerman & Gagne, 2003; Carver et al., 1989), an inventory of coping strategies. The single item: “I drink alcohol or take drugs, in order to think about it less,” was rated on a 4-point Likert scale including 0 (“I usually don’t do this at all”), 1 (“I usually do this a little bit”), 2 (“I usually do this a medium amount”), and 3 (“I usually do this a lot”; see Table 2).

#### 2.3.2. Negative Mood

Negative mood was measured every morning and evening during the 14/15 consecutive days. Nine mood words (e.g., anxious, sad, angry) were selected from the Profile of Mood States (POMS; McNair et al., 1971) to be rated by the participants on a 5-point Likert scale from 0 (“Not at all”) to 4 (“Extremely”). Reliability of individual differences (R_KF_ = 0.99), single time point (R_1F_ = 0.92), and change (R_C_ = 0.87; Shrout & Lane, 2012), were all good to excellent.

#### 2.3.3. Alcohol Use

Alcohol use was measured every evening during the 14/15 consecutive days. Participants rated how many standard drinks they consumed in the past 24 h (“How many standard drinks did you consume in the PAST 24 HOURS?”) on an ordinal scale ranging from 1 (“I did not have anything to drink in the PAST 24 HOURS”) to 17 (“36 drinks or more”). Instructions noted that one “standard drink” is equal to one 12 oz. bottle or can of beer, one 5 oz. glass of wine, or 1 ounce of liquor as a shot or in a mixed drink. Specifically, 1 represented no alcohol consumption in the past 24 h, 2 to 13 represented one to twelve drink(s), respectively, 14 represented thirteen to seventeen drinks, 15 represented eighteen to twenty-three drinks, 16 represented twenty-four to thirty-five drinks, and 17 represented thirty-six and more drinks. Individuals reported drinking on 329 (11.3%) of 2920 evening diary reports. Drinking quantity was highly skewed (*Skew* = 5.82), with 99.8% of reports corresponding to one to ten drinks (1 [*n* = 88], 2 [*n* = 87], 3 [*n* = 45], 4 [*n* = 37], 5 [*n* = 23], 6 [*n* = 15], 7 [*n* = 14], 8 [*n* = 4], 9 [*n* = 2], 10 [*n* = 8]) and only six reports of 13 or more drinks. As a result, we log transformed alcohol use for analyses. Figures depicting frequency of alcohol use by days pre- and post-exam and by weekday are available in the Supplementary Material (Supplementary Figures S1 and S2). As observed in prior studies (Tremblay et al., 2010), alcohol use was more common on Fridays compared to other weekdays (Supplementary Figure S2).

#### 2.3.4. Covariates

In light of the longitudinal nature of the study design spanning two weeks and established differences in negative affectivity and alcohol use as a function of various demographic factors, we included person-level and time-specific covariates to adjust for systematic changes in the focal affect-alcohol associations. At the individual level, we included gender and year in college, as well as daily reports of other substance use, for both alcohol and affect outcomes. In the evening diaries, individuals were also asked to report on their use of other substances (Yes/No), including cigarettes (*n_Yes_* = 150), cannabis (*n_Yes_* = 64), and other recreational drugs (*n_Yes_* = 18). Individually, they showed small positive associations with alcohol use (*r*s = 0.09–0.17) and so were combined into a single variable to adjust for co-use and parallel effects on negative affect. Participant ages were skewed such that the majority of participants were under 21 years of age (191 out of 226). In addition, the categorical correlation coefficient of being over drinking age and year in college was *ϕ* = 0.87, indicating redundancy between the two variables. Thus, year in college, rather than age, was used as a covariate. At the design level, based on initial trajectory analyses for each dependent variable, we included linear effects for day in the study and a spline for post-exam shifts in trajectory. Day in the study was coded such that 0 indicated a report occurred on exam day, with negative values representing pre-exam days and positive values representing post-exam days. Values of this covariate ranged from −8 to 11. The post-exam spline was coded such that 0 indicated that a report occurred on or prior to exam day, with positive values representing post-exam days. Values of this covariate ranged from 0 to 11. We additionally included a dummy indicator for the exam day specifically.

### 2.4. Analytic Plan

Multilevel moderated mediation analyses were conducted to examine the associations among alcohol use, negative affect, coping motives, and the acute stressor of the exam context using SAS PROC MIXED (v9.4; SAS Institute Inc., Cary, NC, USA). The data structure of this study was such that day (level-1) was crossed within person (level-2). Level-1 variables were centered on each individual’s mean and level-2 variables were centered at the grand mean of the sample (Curran & Bauer, 2011). First, between-person and within-person components of morning NA and their interactions with substance use coping were used to predict evening (logged) alcohol use quantity. A random intercept and random slopes, with all random effect correlations, were estimated for the within-person main effects. Next, between-person and within-person components of both morning NA and (logged) evening alcohol use quantity were used to predict evening NA. Similarly, a random intercept and random slopes, with all random effect correlations, were estimated for the within-person main effects. Moreover, given the multivariate construction of the model, we also estimated the random effect correlations between the two sub-model random effects (Bauer et al., 2006).

**Figure 1:**
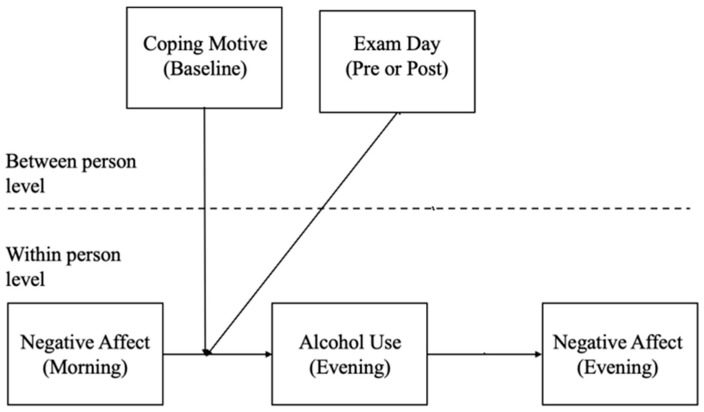
Mediation model depicting the level of measurements.

To examine the condition of acute stress, the exam day variable was dichotomized into pre- and post-exam period representing whether a given measurement occasion was before (“pre-”) or after the exam (“post-”; Figure 1). Exam day was included as a post-exam measurement even though the morning diary was before the exam, because the mediator and outcome assessments were after students had taken their respective exams. We conducted an exploratory analysis of just exam day reports to investigate if there were unique relationships between NA and alcohol when NA reports flanked the occurrence of the acute stressor and we might expect alcohol to be paired with especially strong stress reduction. This analysis did not reveal a distinct set of associations (though was underpowered) and more closely aligned with post-exam results. Covariates were effect coded if categorical and centered if continuous. Given our hypothesis regarding the moderating effect of substance use coping as a person-level characteristic, we only include its interaction with morning NA in predicting alcohol use (*a* path). Additionally, we had no hypotheses regarding pre-exam versus post-exam differences in the association between alcohol use and evening NA (*b* path) and so estimated a single sub-model (Figure 1). We confirmed this choice by estimating a model with separate pre-exam and post-exam associations. There were no statistically significant pre-post differences in any of the associations (*p*s > 0.157), and so the model was collapsed for parsimony.

In addition, we tested a 1–1–1 (all within-level; Bauer et al., 2006) mediation model, using Preacher and Selig (2012) 95% Monte Carlo confidence intervals (MCCIs) in base R (v4.1.2; R Core Team, 2021, Vienna, Austria) to assess statistical significance for the indirect effects.

## 3. Results

Correlations and descriptive statistics between the primary analysis constructs are shown in Table 3, with between-person correlations below the diagonal and within-person correlations above.

**Figure 2:**
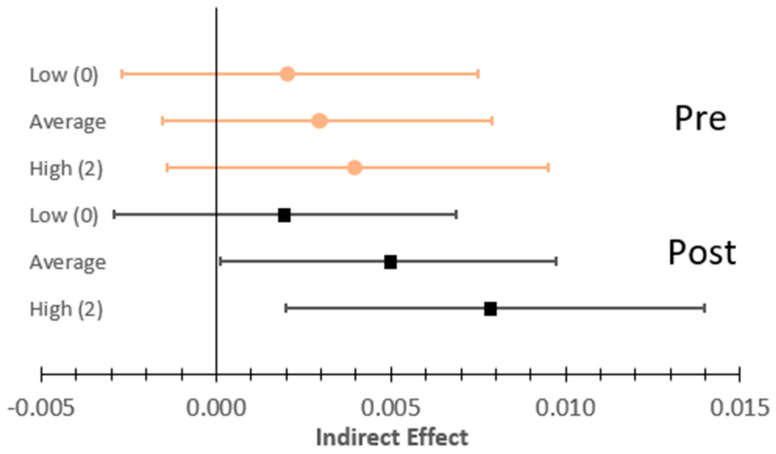
Indirect effects by study phase and level of substance use coping. *Notes*. Pre-exam (circles, orange) and Post-exam (squares, black) indirect effect estimates at low (0), average, and high (2) reported substance use coping. Squares or circles represent standardized (β) point estimates, with the boundaries of the lines representing the 95% confidence interval for each point estimate.

Table 4 presents the results for the full multilevel mediation analysis. Inconsistent with the first hypothesis, prior to the exam, individuals did not endorse greater alcohol use on days when they had greater morning NA (*β* = −0.034, *SE* = 0.025, *p =* 0.171) or if they experienced more NA overall (*β* = 0.021, *SE* = 0.034, *p =* 0.531). And substance use coping did not moderate either of these effects (*β*s < |0.020|, *p*s *>* 0.488). However, consistent with the first hypothesis, both prior to and after the exam, days with higher alcohol use were associated with lower evening negative affect (*β* = −0.043, *SE* = 0.012, *p <* 0.001). Overall, the indirect effect of morning to evening NA reductions through increased alcohol use was not observed prior to the exam (Figure 2), ostensibly when individuals were under increased stress.

In contrast to the second hypothesis, baseline coping motives were more positively associated with evening alcohol use following the exam than prior to it (*β*_Post-Pre_ = 0.118, *SE* = 0.039, *p =* 0.003). Consistent with the second hypothesis, after the exam, and thus following the period of acute stress, individuals who experienced greater morning negative affect than usual tended to use less alcohol (*β* = −0.074, *SE* = 0.022, *p =* 0.001). This post-exam negative effect was magnified for those who reported greater substance use coping (*β* = −0.068, *SE* = 0.023, *p =* 0.004). This pattern of findings was also observed at the between-person level, though the effects were smaller and not statistically significant.

An indirect effect operating through alcohol use was observed for the weeklong period after the exam; however, it did not serve to reduce elevated levels of negative affect. Similar to the pre-exam period, individuals who reported that they did not endorse any substance use coping did not experience any changes in NA through the use of alcohol (*β_Ind_* = 0.0019, 95% *CI* = [−0.0029, 0.0068]). Individuals who reported any substance use coping (*β_Ind_* = 0.0048, 95% *CI* = [0.0001, 0.0097]), but especially those who endorsed medium (“I usually do this a medium amount,” *n* = 12) or high (“I usually do this a lot,” *n* = 2) levels (*β_Ind_* = 0.0077, 95% *CI* = [0.0002, 0.0140]), consumed less alcohol than they typically did in response to elevated morning NA, which contributed to them experiencing even more NA in the evening than they did in the morning. Conversely, this effect implies that individuals who experienced less morning NA than typical were then likely to consume more alcohol, further reducing their evening NA compared to morning.

Consistent with research showing differences in the effects of stress (Patock-Peckham et al., 2022) and coping (Cooper et al., 1992) on alcohol use by gender, gender significantly predicted alcohol use (*β* = −0.068, *SE* = 0.025, *p* = 0.006; Supplementary Table S2). Models separately estimated by gender found that overall effects were consistent with effects among women (Supplementary Table S2). In contrast to women, men who on average experienced more NA and endorsed higher coping motives reported lower alcohol use prior to the exam and higher alcohol use after the exam (Supplementary Table S4). Higher than usual negative affect was not associated with changes in drinking, endorsing drinking to cope did not predict alcohol use pre-exam, and alcohol use did not predict NA relief as in women (Supplementary Table S4). Further detail on effects of gender and other covariates are available in the Supplementary Material (Supplementary Table S1).

## 4. Discussion

This study investigated whether alcohol use predicts negative affect relief in a sample undergoing an acute stressor, and whether this relationship is affected by coping motives. Alcohol use was found to mediate the relationship between morning and evening negative affect in a sample of college students during a period of acute stress; however, contrary to predictions, only after the stressful event, and not in the predicted direction. In support of our predictions, we detected a within-person effect such that evening negative affect decreased as alcohol use increased, both during the period leading up to and period after the acute stressor. Although we did not find a difference in this effect pre- and post-acute stressor, prior research that has not operationalized acute stress has found no relationship between substance use and changes in self-reported mood (Wycoff et al., 2021). In addition, although college students have reported using alcohol in the context of celebrations (e.g., holidays; Tremblay et al., 2010), they have also reported using less alcohol during exams (Noel & Cohen, 1997; Tremblay et al., 2010). Thus, the post-exam period in this study, rather than being an immediate return to exam-free baseline, may instead encompass the recovery period of the acute stress response (Sinha, 2024). This may indicate that acute stress and its recovery period increase the likelihood that alcohol use will lead to improvement in mood.

In contrast to our predictions, an indirect effect of morning negative affect on evening negative affect via alcohol use was not detected during the pre-exam period. Rather an indirect effect was observed during the post-exam period, such that higher morning negative affect predicted lower evening alcohol use, which in turn predicted higher evening negative affect. In particular, this relationship existed among individuals who endorsed moderate to high levels of substance use coping. It should be noted that this mediation test is limited by the fact that evening negative affect and alcohol use were measured simultaneously rather than measurement of affect preceding measurement of alcohol use. However, this pattern, which partially supports but also adds nuance to theories of addiction that emphasize negative affect relief as a common driver of alcohol use, has not always been detected in other intensive longitudinal studies and to an extent reflects the more common ‘mixed’ support (Dora et al., 2023). It is congruent though with prior findings that alcohol consumption may reduce the impact of stress on internalizing symptoms (Révész et al., 2022; Lipton, 1994). Our findings suggest that these widely theorized relationships may not necessarily emerge during acutely stressful periods and may manifest differently post-stress when there is still uncertainty about the outcome.

That is, a different indirect effect of morning negative affect on evening negative affect via alcohol use emerged among individuals high in substance use coping during the post-exam period. In this case, evening drinking decreased as morning negative affect increased, and evening negative affect increased as evening drinking decreased. In other words, for individuals high in coping motives, decreased drinking following the exam was associated with increased negative affect later in the day. When combined with the pre-exam results, this indicates that, at least among some individuals, alcohol use effectively downregulated negative affect prior to a stressful event, and reduced alcohol use after the event was associated with a simultaneous increase in negative affect. Somewhat surprisingly, however, this model also indicated that once the acute stressor was removed, individuals high in coping motives consumed less alcohol on days that began with higher negative affect. Individuals who endorse coping motives for alcohol use may perceive themselves as drinking more when negative affect is high to cope with an acute stressor. In fact, they may transition from drinking less in response to negative affect when stress is low to drinking in similar quantities regardless of affect when stress is high. This finding would be consistent with research showing discrepancies between subjective relief due to alcohol use and actual changes in affect following alcohol use (Wycoff et al., 2021). In addition, other authors have argued that alcohol use may be used as a coping response in some situations but not in others (Dora et al., 2023).

Future studies may examine whether this pattern of results explains some of the ambiguity in the literature on the relationship between negative affect and alcohol use in daily life, such that among individuals who endorse coping motives (Stanesby et al., 2019) or their associated personality characteristics (Kuntsche et al., 2006) the relationship between negative affect and alcohol use undergoes a change during periods of stress. In terms of the impact of coping motives on the association between stress and alcohol use, our predictions were not supported. Within participants after the exam and between participants both before and after the exam, the relationship between morning negative affect and alcohol use decreased as coping motives increased. In other words, after the exam, individuals who endorsed higher coping motives consumed less alcohol on days when they experienced higher morning negative affect. In addition, those who tended to have higher morning negative affect reported less alcohol use after the exam if their baseline coping motives were higher.

Although there is considerable research indicating that coping motives predict alcohol use, and indeed, the main effect of coping motives on alcohol use was positive and significant both before and after the exam, less consideration has been given to the role of coping motives in the relationship between negative affect and alcohol use. Coping motives may not exist outside of the causal relationships between affect, alcohol use, and later affect, as would typically be required from a moderating variable (Kraemer et al., 2002). Although measured as a baseline characteristic in the present study, other research has identified daily variance in coping motives and has shown them to be associated with negative affect (Biehler et al., 2024). One explanation for these unexpected effects, therefore, might be that the measures used in our study did not allow bi-directional relationships between coping motives and other constructs of interest to be tested. In addition, expanding consideration from only coping motives to the impact of coping style broadly on alcohol use may be beneficial, as overall coping style has repeatedly been shown to impact the relationship between stress and alcohol-related outcomes, even to the point of at times reversing their relationship (Veenstra et al., 2007). In addition, several studies have found sex differences in the effect of stress on alcohol use (e.g., Patock-Peckham et al., 2022). In this study, men who reported more NA on average and higher coping motives reported less alcohol consumption prior to the exam and more alcohol consumption after the exam. This may reflect that self-awareness regarding drinking to cope is protective, particularly among men who may be higher in anxiety and therefore possibly prevention-focus (Klenk et al., 2011). However, both significant and non-significant differences in results by gender in this sample should be interpreted with caution, as women comprised a substantial majority of the sample.

In addition to coping motives, foundational work (Cooper, 1994; Cox & Klinger, 1988) has led researchers to measure drinking motives including desire to increase positive social experiences (social motives), desire to upregulate positive affect (enhancement motives), and desire to decrease negative social experiences (conformity motives). Social and enhancement motives are more common than coping motives, at least among young adults (Kuntsche et al., 2005). Moreover, high endorsement of enhancement motives has been linked to heavy drinking, particularly among women in predominantly male social contexts (Stanesby et al., 2019). Thus, alcohol use surrounding acute stressors is likely impacted not only by students’ desire to reduce negative affect associated with stress but also by their social environment and their motivation to increase positive affect.

Furthermore, it should be noted that stress has been shown to increase alcohol craving (Wemm et al., 2022), and the analyses reported here do not disaggregate negative affect associated with alcohol craving from negative affect resulting directly from the stressor. Repeated alcohol use increases sensitivity to alcohol-related cues (Cofresí et al., 2019), and among individuals who regularly consume more alcohol, craving predicted by stress positively predicts alcohol use (e.g., Wemm et al., 2022; Blaine et al., 2019). Thus, rather than representing an attempt to cope with negative affect precipitated by stress, some of the alcohol use observed in this sample may result from stress-induced craving. In addition, the increase in negative affect via reduced alcohol use observed post-exam among individuals who endorsed substance use coping could be explained by alcohol reducing not only general negative affect, but specifically craving, especially among those who typically drink more alcohol (Treloar & Miranda, 2017).

Finally, the hypothesis that previously predicted day-level relationships between negative affect and alcohol use would hold under conditions of acute stress, but not afterward, was partially supported. Alcohol use was associated with reduced evening negative affect before and after the exam. In addition, at the between-person level, greater morning negative affect after the exam was marginally associated with less alcohol use rather than more. Coping motives, however, did not impact the relationship between stress and alcohol use in the expected direction prior to an exam compared to after it.

## 5. Limitations and Future Directions

Future studies should continue to examine the conditions under which stress evokes theorized relationships between negative affect and alcohol use. Worth consideration is social support, which is positively associated with alcohol use (Veenstra et al., 2007). In addition, seeking social support to cope is also positively associated with use (Aldridge-Gerry et al., 2011). Future research should examine whether seeking social support increases likelihood of drinking not because alcohol use in itself is expected to relieve negative affect, but because the social situations pursued involve alcohol, consistent with research on social motives for alcohol use (Cooper, 1994). In addition, developing evidence regarding the role of expectancies in the relationship between stress and alcohol use may clarify the relative role of motives, as alcohol use arising simply from willingness rather than motivation to use may explain some of the increase in alcohol use during stressful periods (Armeli et al., 2000; Anderson et al., 2017).

Because repeated alcohol use is believed to lower individuals’ average mood, further scrutiny of the effect of between-subjects differences in mood is warranted (Anthenelli, 2012). For example, in the present study, though person-level individual differences in morning negative affect did not predict evening alcohol use prior to an exam, higher levels of negative affect were associated with marginally decreased alcohol use after the exam. It is possible that moving from less alcohol consumed on days with higher negative emotion to the same amount of alcohol consumed under conditions of higher stress may be subjectively experienced as drinking more when under stress. Future research may also examine factors that protect individuals from the relationship between negative affect and alcohol use during times of stress. Components of self-compassion, for example, have been shown to have correlations with negative affect and coping motives, though this study did not find interaction effects, perhaps due to low statistical power (Biehler et al., 2024). Finally, one promising avenue is the interaction of stress typology and alcohol use. Some research indicates that the nature of the stressor may alter its impact on alcohol use (Sinha, 2024). For example, women may be less likely to drink under conditions of daily life stress (Breslin et al., 1995), and they experience higher rates of certain types of early life stress, which is associated with alcohol use disorder (Guinle & Sinha, 2020). Consistent with Patock-Peckham et al. (2022) we also agree that the impact of sex should continue to be explored.

Moreover, it is important to consider the context and sample characteristics in which this study was conducted. Much of the sample was under the legal drinking age. However, the average age of 20.0 in the sample is also promising in terms of generalizability to individuals with alcohol-related problems, as young adults ages 18–29 are most likely to develop AUD (Glantz et al., 2020). The college undergraduates recruited were enrolled at highly competitive, private universities where academic performance motivation would be assumed to be very high. As observed in the data, substance use coping was rarely endorsed at higher levels and drinking behavior was comparatively low in terms of frequency (11% of days) and quantity (53% were 1–2 drinks), despite over half the sample reporting drinking during the diary period. As a result, few individuals in the sample might be expected to be in even prodromal stages of AUD risk where NA might potentiate alcohol use in response to stress (Sher & Levenson, 1982) and instead may be more likely to be highly regulated, utilize other coping strategies, or even hold negative attitudes towards behaviors that may undermine achievement. Lastly, the study used a convenience sample composed of 70% female participants, which may limit the generalizability of the findings.

Although study measurement and statistical analyses had numerous strengths including enabling disaggregation of within- and between-person effects, they were also limited in several ways. For example, in this study, there was no individual-level rating of the degree to which participants perceived the exam to be stressful, which could be tested in future studies as a prospective predictor of negative affect. Moreover, the stress experienced by individuals might vary depending on their academic goals, such that for those who were not pre-med majors, they might experience less stress as their performance on the exam did not carry the same implications as it did for those pursuing medical school. Also, tendency to engage in substance use coping was measured using a single item from a background measure based on a well-known coping scale (the revised COPE; Zuckerman & Gagne, 2003; Carver et al., 1989). Measurement options were limited by the nature of this study as a secondary data analysis, and single-item measures of alcohol use coping are not uncommon in the literature (e.g., Mauro et al., 2015). However, multi-item measures are often recommended over single-item measures of psychological constructs because researchers can calculate the internal consistency of a multi-item scale (Wanous & Reichers, 1996). The use of a single item measure of coping therefore constitutes a limitation of this study. Future studies should consider multi-item measurements of alcohol use coping such as the coping subscale of the Drinking Motives Questionnaire (Cooper, 1994).

In addition, participants were asked to report their standard drinks in the past 24 h in their evening reports. As a result, bias may have been introduced if participants answered their evening surveys at different times on different days, leading to responses that included overlapping temporal periods. Future studies should consider wording that minimizes such overlap (e.g., “…since your last evening report”). Moreover, our study did not assess mood more proximally before alcohol use, for example in the afternoon or early evening. Such assessments would align more closely with theorized timescales and might have yielded different (hypothesized) effects, especially in the pre-exam period. Finally, although higher levels of substance use coping motives had a stronger effect on the indirect effect of morning NA on evening NA via alcohol use during the post-exam period, it should be noted that only *n* = 14 individuals endorsed medium or high levels of substance use coping. Thus, the findings here may not generalize to all individuals who would endorse medium or high levels of substance use coping.

## 6. Conclusions

The current investigation sought to evaluate whether the first step in the long-proposed negative reinforcement cycle of alcohol use, whereby negative affectivity motivates use for relief, is observed, and is successful at reducing negative feelings. Though anticipated, the experience of an acute stressor did not lead negative affect to predict use, nor did use alleviate such negative affect. Rather, during a recovery period, when the outcome of the stressor might still have been unknown, negative affect was associated with less drinking, potentially as a regulatory effort, but drinking was associated with less negative affect. These associations suggest a different, perhaps complementary, “positive” side of the negative reinforcement process where feeling less negative is itself a signal that it is safer to engage in otherwise risky drinking behavior, and the effects of drinking then serve to further reduce already lower initial negative feelings.

## Figures and Tables

**Table 1:** Participant characteristics (categorical).

	*N*	*%*
Gender		
Female	160	70.8
Male	66	29.2
Other Identity	0	0.0
Sexual Identity		
Straight	209	93.3
Gay or Lesbian	6	2.7
Bisexual	6	2.7
Other	3	1.3
Race/Ethnicity/Identity		
Asian	99	43.8
White	97	42.9
Black	15	6.6
Native American	6	2.7
Pacific Islander	3	1.3
Hispanic	29	13.0
International	63	27.9
Other	28	12.4
Year in College		
Freshman	72	31.9
Sophomore	76	33.6
Junior	45	19.9
Senior	13	5.8
Other	20	8.9
Exam Course		
Chemistry	125	55.3
Biology	75	33.2
Physics	26	11.5

**Table 2:** Participant characteristics (continuous).

	*N*	*%*	*M*	*SD*	*Range*
Age	226	100.0	20.0	2.1	18–29
Drinking Days	226	100.0	1.5	2.1	0 (108), 1 (40), 2 (23), 3 (24), 4 + (31)
Drinks per Drinking Day	118	52.2	3.3	2.7	1–17
Other Substance Use Days	226	100.0	0.9	2.6	0 (175), 1 (16), 2 (12), 3 (4), 4 + (19)
Substance Use Coping	226	100.0	0.3	0.6	0 (179), 1 (33), 2 (12), 3 (2)

*Notes*. Range values in parentheses are the number of individuals at each value. “Drinking Days” refers to days of drinking per participant across their entire daily diary period of 14–15 days.

**Table 3:** Between- and within-person bivariate correlations.

		*M*	*SD_Btw_*	*SD_Wth_*	1.	2.	3.	4.
1.	Coping	0.28	0.60	–		–	–	–
2.	# Drinks per Day	0.39	0.61	1.25	0.33 ***		−0.05 *	−0.04 *
3.	Morning NA	0.71	0.62	0.50	0.27 ***	−0.02		0.34 ***
4.	Evening NA	0.74	0.63	0.52	0.26 ***	−0.04	0.95 ***	

*Notes*. NA = negative affect, *SD_Btw_* = between-person standard deviation, *SD_Wth_* = within-person standard deviation. # connotes number. * *p* < 0.05, *** *p* < 0.001. Between-person correlations are displayed below and within-person correlations above the diagonal.

**Table 4:** Multilevel moderated mediation model parameter estimates.

	Pre-Exam			Post-Exam			Post-Pre Difference		
Effect	β	*SE*	*p*	β	*SE*	*p*	β	*SE*	*p*
ALCOHOL USE									
Intercept	0.308	0.041	<0.001	0.440	0.037	<0.001	0.132	0.036	<0.001
Coping	0.086	0.037	0.020	0.204	0.033	<0.001	0.118	0.039	0.003
Within									
Morning NA	−0.034	0.025	0.171	−0.074	0.022	0.001	−0.039	0.034	0.247
Morning NA * Coping	−0.018	0.026	0.488	−0.068	0.023	0.004	−0.050	0.036	0.162
Between									
Morning NA	0.021	0.034	0.531	−0.048	0.030	0.105	−0.069	0.037	0.063
Morning NA * Coping	−0.014	0.024	0.542	−0.038	0.022	0.080	−0.024	0.027	0.377
EVENING NA									
Intercept	1.009	0.030	<0.001	1.009	0.030	<0.001			
Within									
Morning NA	0.230	0.016	<0.001	0.230	0.016	<0.001			
# Drinks	−0.043	0.012	<0.001	−0.043	0.012	<0.001			
Between									
Morning NA	0.728	0.016	<0.001	0.728	0.016	<0.001			
# Drinks	0.024	0.017	0.165	0.024	0.017	0.1645			
Within Indirect Effect	β	5%	95%	β	5%	95%			
Low (0) Coping	0.0024	−0.0027	0.0075	0.0019	−0.0029	0.0068			
Average Coping	0.0031	−0.0015	0.0079	0.0048	0.0001	0.0097			
High (2) Coping	0.0039	−0.0014	0.0095	0.0077	0.0020	0.0140			

*Notes*. SE = standard error, NA = negative affect. Coefficients estimated by standardizing each variable by its overall standard deviation after disaggregation in order to preserve estimable individual differences in the random effects. * connotes an interaction, # connotes number, and highlighting connotes significant indirect effects and the main effects that contribute to them.

## Data Availability

Data used in this study are available from the authors upon request. Requests will be reviewed by the authors to ensure that the rights and privacy of research subjects are preserved.

## Supplementary Materials

The following supporting information can be downloaded at: https://www.mdpi.com/article/10.3390/bs15121614/s1, Table S1: Exam results; Table S2: Multilevel moderated mediation model parameter estimates with covariates included; Table S3: Multilevel moderation model parameter estimates for women; Table S4: Multilevel moderation model estimates for men; Figure S1: Percentage of alcohol use reports pre- and post-exam; Figure S2: Count and percentage of alcohol use reports by weekday.
