# Supplementary material for: Efficacy of Coping with Negative Affect via Alcohol Use Pre- and Post Acute Stress
Source: Behav Sci (Basel). 2025 Nov 24;15(12):1614. doi: 10.3390/bs15121614 (PMC12729561; doi:10.3390/bs15121614)
Supplement: Supplementary file 1 [file behavsci-15-01614-s001.zip › behavsci-3820704-supplementary.pdf]

Supplemental Materials

Supplementary Table S1. Exam results.

Letter Grade	*N*
A	39
A-	23
B+	19
B	25
B-	17
C+	11
C	12
C-	2
D+	5
D	7
F	6

Supplementary Table S2. Multilevel moderated mediation model parameter estimates with covariates included

	**Pre-Exam**			**Post-Exam**			**Post-Pre Difference**		
Effect	β	*SE*	*p*	β	*SE*	*p*	β	*SE*	*p*
**ALCOHOL USE**									
Intercept	0.308	0.041	<.001	0.440	0.037	<.001	0.132	0.036	<.001
Coping	0.086	0.037	.020	0.204	0.033	<.001	0.118	0.039	.003
Within									
Morning NA	-0.034	0.025	.171	-0.074	0.022	.001	-0.039	0.034	.247
Morning NA * Coping	-0.018	0.026	.488	-0.068	0.023	.004	-0.050	0.036	.162
Between									
Morning NA	0.021	0.034	.531	-0.048	0.030	.105	-0.069	0.037	.063
Morning NA * Coping	-0.014	0.024	.542	-0.038	0.022	.080	-0.024	0.027	.377
Covariates									
Gender (1 = female)	-0.068	0.025	.006	-0.068	0.025	0.006			
Year in college	0.119	0.025	<.001	0.119	0.025	<.001			
Other substance use	0.181	0.020	<.001	0.181	0.020	<.001			
**EVENING NA**									
Intercept	1.009	0.030	<.001	1.009	0.030	<.001			
Within									
Morning NA	0.230	0.016	<.001	0.230	0.016	<.001			
# Drinks	-0.043	0.012	<.001	-0.043	0.012	<.001			
Between									
Morning NA	0.728	0.016	<.001	0.728	0.016	<.001			
# Drinks	0.024	0.017	.165	0.024	0.017	.165			
Covariates									
Exam day (-8 to 11)	0.082	0.031	.009	0.082	0.031	.009			
Exam on day of report	-0.090	0.013	<.001	-0.090	0.013	<.001			
Days post-exam (0 to 11)	-0.115	0.032	<.001	-0.115	0.032	<.001			
Gender (1 = female)	0.018	0.016	.257	0.018	0.016	.257			
Year in college	-0.016	0.016	.322	-0.016	0.016	.322			
Other substance use	-0.005	0.014	.724	-0.005	0.014	.724			

Within Indirect Effect	β	5%	95%	β	5%	95%
Low (0) Coping	0.0024	-0.0027	0.0075	0.0019	-0.0029	0.0068
Average Coping	0.0031	-0.0015	0.0079	0.0048	0.0001	0.0097
High (2) Coping	0.0039	-0.0014	0.0095	0.0077	0.0020	0.0140

Supplementary Table S3. Multilevel moderation model parameter estimates for women.

	**Pre-Exam**			**Post-Exam**			**Post-Pre Difference**		
Effect	β	*SE*	*p*	β	*SE*	*p*	β	*SE*	*p*
**ALCOHOL USE**									
Intercept	0.231	0.044	<.001	0.365	0.040	<.001	0.134	0.042	.002
Coping	0.093	0.042	.029	0.194	0.037	<.001	0.102	0.045	.022
Within									
Morning NA	-0.022	0.030	.466	-0.095	0.025	<.001	-0.073	0.040	.067
Morning NA * Coping	-0.031	0.029	.283	-0.063	0.025	.011	-0.032	0.039	.403
Between									
Morning NA	0.006	0.040	.877	-0.065	0.034	.057	-0.071	0.043	.096
Morning NA * Coping	-0.000	0.026	.994	-0.052	0.023	.027	-0.051	0.029	.074
**EVENING NA**									
Intercept	1.030	0.034	<.001	1.030	0.034	<.001			
Within									
Morning NA	0.226	0.018	<.001	0.226	0.018	<.001			
# Drinks	-0.056	0.014	<.001	-0.056	0.014	<.001			
Between									
Morning NA	0.718	0.018	<.001	0.718	0.018	<.001			
# Drinks	0.032	0.023	.157	0.032	0.023	.157			

Notes. SE = standard error, NA = negative affect. Coefficients estimated by standardizing each variable by its overall standard deviation after disaggregation in order to preserve estimable individual differences in the random effects.

Supplementary Table S4. Multilevel moderation model estimates for men.

	**Pre-Exam**			**Post-Exam**			**Post-Pre Difference**		
Effect	β	*SE*	*p*	β	*SE*	*p*	β	*SE*	*p*
**ALCOHOL USE**									
Intercept	0.397	0.084	<.001	0.514	0.078	<.001	0.118	0.072	.104
Coping	-0.012	0.083	.882	0.279	0.075	<.001	0.291	0.083	.001
Within									
Morning NA	-0.026	0.049	.596	0.016	0.049	.742	0.042	0.071	.550
Morning NA * Coping	0.084	0.067	.213	-0.074	0.069	.285	-0.158	0.098	.109
Between									
Morning NA	-0.001	0.076	.993	0.019	0.068	.785	0.019	0.083	.816
Morning NA * Coping	-0.264	0.094	.005	0.208	0.077	.007	0.471	0.100	<.001
**EVENING NA**									
Intercept	0.998	0.059	<.001	0.998	0.059	<.001			
Within									

Morning NA	0.244	0.031	<.001	0.244	0.031	<.001
# Drinks	-0.019	0.020	.327	-0.019	0.020	.327
Between						
Morning NA	0.759	0.036	<.001	0.759	0.036	<.001
# Drinks	0.041	0.027	.133	0.041	0.027	.133

Notes. SE = standard error, NA = negative affect. Coefficients estimated by standardizing each variable by its overall standard deviation after disaggregation in order to preserve estimable individual differences in the random effects.

Supplementary Figure S1. Percentage of alcohol use reports pre- and post-exam.

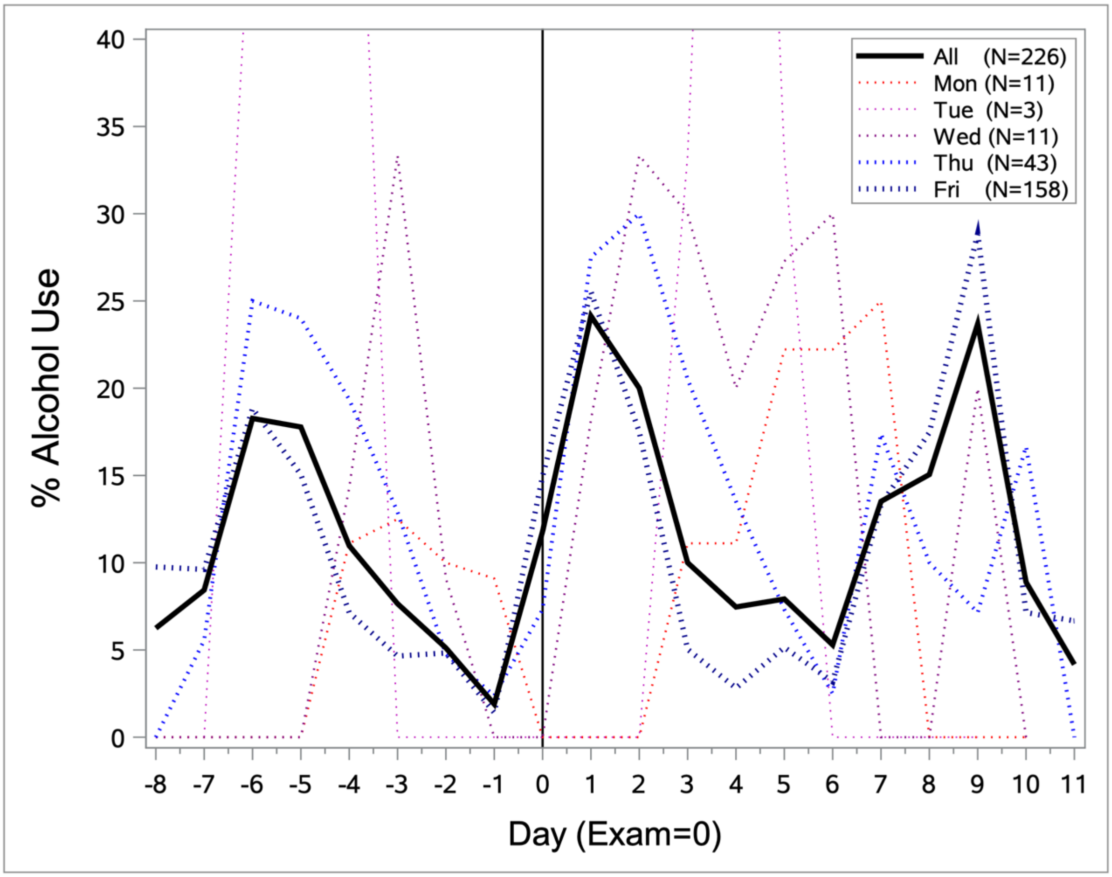

Supplementary Figure S2. Count and percentage of alcohol use reports by weekday.

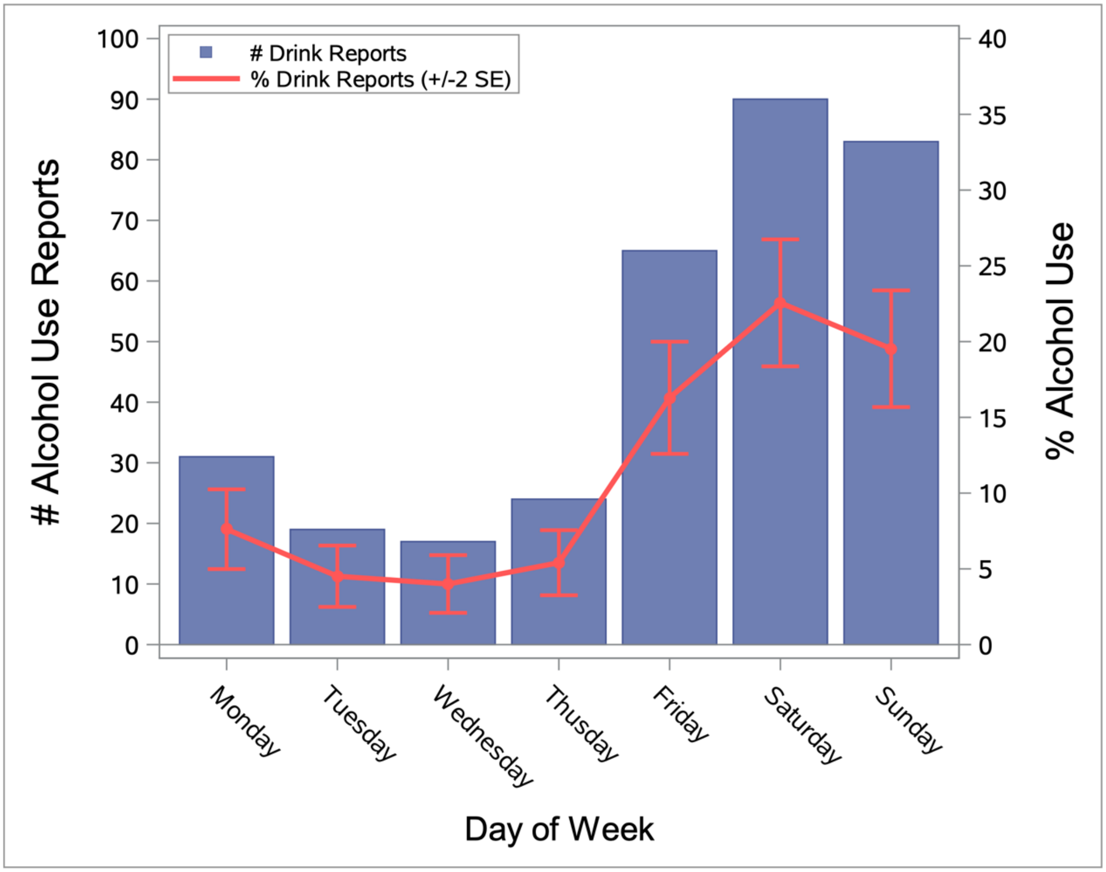

Drink Reports
% Drink Reports (+/-2 SE)
# Alcohol Use Reports
% Alcohol Use
100
90
80
70
60
50
40
30
20
10
0
40
35
30
25
20
15
10
5
0
Monday
Tuesday
Wednesday
Thusday
Friday
Saturday
Sunday
Day of Week
